# Comparative evaluation of two commercial ELISA kits for detection of antibodies against Akabane virus in cattle serum

**DOI:** 10.1186/s12917-019-2156-6

**Published:** 2019-11-11

**Authors:** Xiaolin Li, Hongli Jing, Xiaofei Liu, Qin Wang, Songyin Qiu, Dandan Liu, Shaoqiang Wu, Xiangmei Lin

**Affiliations:** Institute of Animal Quarantine, Chinese Academy of Inspection and Quarantine, Beijing, 100176 China

**Keywords:** Anti-AKAV antibody, Diagnostic sensitivity, Diagnostic specificity, Virus neutralization test

## Abstract

**Background:**

Akabane disease (AD), a barrier to international trade for endemic areas with far economic impact on the countries, is caused by Akabane virus (AKAV). Commercial enzyme-linked immunosorbent assay (ELISA) is a commonly used diagnostic technique for AKAV infection, including the IDEXX and IDVET ELISA kits. However, the comparative evaluation of the IDEXX and IDVET ELISA kits has not been published. The object of this study was to evaluate the test performance of the two commercial ELISA kits in detecting serum anti-AKAV antibodies in cattle.

**Results:**

With virus neutralization test (VNT) as the “relative gold standard”, the diagnostic sensitivity (DSe) was 80.39% (123/153) and 93.46% (143/153) for the IDEXX and IDVET ELISA kit, when suspect samples were included. The diagnostic specificity (DSp) for the IDEXX and IDVET ELISA kit was 93.48% (502/537) and 82.31% (442/537), respectively.

**Conclusion:**

Both of the tested ELISA kits could be applied to detect antibodies against AKAV in cattle serum. The IDVET ELISA kit had a higher DSe. The IDEXX ELISA kit possessed the higher DSp. These results have important implications if the kits are used to screen herds or individual cattle in surveillance programs, or at border crossings for import-export inspection and quarantine.

## Background

Akabane disease (AD), characterized by abortions, stillbirth and congenital defects in pregnant ruminants, is caused by AKAV which was first identified by virus isolation in Japan [1]. Nowadays, AD has been a barrier to international trade for endemic areas, with far economic impact on the countries. To date, AD has been found in cattle and sheep in Australia, Asia, the Middle East and Africa [2, 3].

AKAV is a member of the Simbu serogroup belonging to the genus Orthobunyavirus in the family *Peribunyaviridae* (order *Bunyavirales*) [4–6], which also includes Schmallenberg virus (SBV) [7], Shamonda virus (SHAV) [8], Douglas virus (DOUV) [9] and Sathuperi virus (SATV) [10]. Some strains of AKAV can cause encephalomyelitis in calves and adult cattle [11, 12]. AKAV is an arthropod-borne virus with a negative-stranded tripartite RNA genome comprising large (L), medium (M), and small (S) segments. The M segment encodes the viral surface glycoproteins (G1 and G2), which participate in the induction of neutralizing antibodies. The S segment encodes a nucleocapsid (N) and a non -structural (NSs) protein. N protein, a group reactive antigen, is able to react with antibodies elicited by other viruses belonging to the serogroup [13–15]. The conserved antigenicity of N protein has been found in many reassortants [16, 17].

Diagnosis of infections caused by AKAV is traditionally accomplished by detection of specific antibodies through virus neutralization test (VNT), and if necessary, the virus is identified by virus isolation. These techniques are labor-intensive, time-consuming and difficult to implement for large numbers of samples [18]. Several commercial ELISAs have been developed to detect antibodies against AKAV, which are ready-to-use and can be applied to large-scale screening and serological investigations [19–21]. A competition ELISA kit was developed by ID.vet Innovative Diagnostics (IDVET ELISA). It was coated with purified AKAV virus and was designed to detect antibodies against AKAV in serum or plasma samples from cattle, sheep and goats. Another commercial kit, coated with purified SBV N protein, was an indirect ELISA kit from IDEXX Laboratories, Inc. (IDEXX ELISA). It was used to detect SBV and other Simbu serogroup viruses in serum and plasma samples from cattle, sheep and goats. However, there is no report on the comparative evaluation of the two ELISA kits for the detection of antibodies to AKAV.

The purpose of the present study was to evaluate the diagnostic performance of two frequently used commercial ELISA kits in detecting anti-AKAV antibodies in cattle serum samples, with the aim to determine the ELISA kit that would be suitable for AKAV surveillance programs or in the process of import-export inspection and quarantine.

## Results

### Detection of anti-AKAV antibodies using VNT, and two ELISA kits

The AKAV infection status of the 690 bovine serum samples used in this study was determined by VNT. Of the sera tested, 153 (22.17%) were positive and 537 (77.83%) were negative (Table 1). The LOD was 1/64, 1/4 and 1/8 for R521, 93,124 and 5188, respectively (Table 2).

**Table 1:** The DSe, DSp and kappa coefficient (κ) of the IDEXX and IDVET ELISA kit compared to VNT as “gold standard”

VNT	IDEXX ELISA kit		IDVET ELISA kit	
	positive	negative	positive	negative
positive	123	30	143	10
negative	35	502	95	442
DSe	80.39% (123/153)		93.46% (143/153)	
DSp	93.48% (502/537)		82.31% (442/537)	
κ value	0.730		0.632

**Table 2:** The LOD of the IDVET ELISA kit (A), the IDEXX ELISA kit(B), and VNT

Dilution of sera	Sera number								
	R521			93,124			5188		
	VNT	A	B	VNT	A	B	VNT	A	B
1:1	+	+	+	+	+	+	+	+	+
1:2	+	+	+	+	+	+	+	+	–
1:4	+	+	+	+	+	+	+	+	–
1:8	+	+	–	–	–	–	+	+	–
1:16	+	+	–	–	–	–	–	–	–
1:32	+	+	–	–	–	–	–	–	–
1:64	+	+	–	–	–	–	–	–	–
1:128	–	–	–	–	–	–	–	–	–
1:256	–	–	–	–	–	–	–	–	–

There was a “grey zone” for data interpretation when using the IDEXX ELISA kit (S/*P* < 30%: negative; S/*P* ≥ 40%: positive and S/*P* between 30 and 40%: inconclusive), we used the 30% as the cut-off of a positive result (i.e. an S/*P* ≥ 30% were scored as positive). Using this criterion, 158 (22.89%) of the tested samples were identified as positive and 532 (77.11%) were identified as anti-AKAV antibody negative. There were 15 samples in the grey zone as defined by the kit criteria but these were classified as positive for this study. For the R521, 93,124 and 5188, the IDEXX ELISA kit could detect a dilution of 1/4, 1/4 and 1/1.

The IDVET ELISA kit also had a “grey zone” for data interpretation (S/*N* < 30%: positive; S/*N* ≥ 40%: negative and S/*N* between 30 and 40%: inconclusive); in this kit, we used the 40% as the cut-off of a positive result (i.e. an S/*N* value of < 40% was considered positive). Using this criterion, 238 (34.49%) of the serum samples were considered positive, while 452 (65.51%) were considered anti-AKAV antibody negative. There were 79 samples in the grey zone as defined by the kit criteria but these were classified as positive in our study. The 1/64, 1/4 and 1/8 of R521, 93,124 and 5188 could be detected, respectively.

### Comparison of the IDEXX ELISA kit, the IDVET ELISA kit, and VNT

**Fig. 1:**
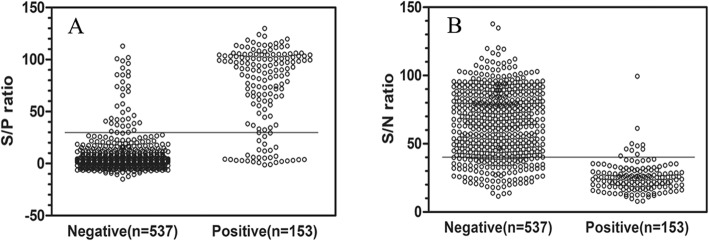
Distribution of ELISA kit results by sample classifications (negative, positive) relative to the assay cutoff. **a**. Results from the IDEXX ELISA kit (sample-to-positive [S/*P*] ratio = 30). **b**. Results from the IDVET ELISA kit (sample-to-positive [S/*N*] ratio = 40)

For the IDEXX ELISA kit, 625 samples gave the same results as when tested by VNT, with 123 positive and 502 negative sera from the 690 serum samples tested. The distribution of S/*N* ratios by sample classification (negative, positive) was given in Fig. 1a. This gave an κ value of 0.730, demonstrating a substantial concordance between the two methods (the IDEXX ELISA kit and VNT). The results showed that this ELISA had a high DSp (94.38%) and DSe (80.39%) at the recommended cutoff.

Results from the IDVET ELISA kit revealed 583 serum samples with consistent results between the ELISA kit and VNT, which included 143 positive and 442 negative samples (Table 1). The S/*P* ratios for all defined samples were shown in Fig. 1b. The κ value between the IDVET ELISA kit and VNT was 0.632, indicating a substantial concordance. At the recommended cutoff, the ELISA kit possessed a DSp of 82.31% and a high DSe of 93.46%. Same with the results of VNT, the LOD of the IDVET ELISA kit showed a slightly higher than that of the IDEXX ELISA kit.

### The ROC analysis of the IDEXX and IDVET ELISA kit

**Fig. 2:**
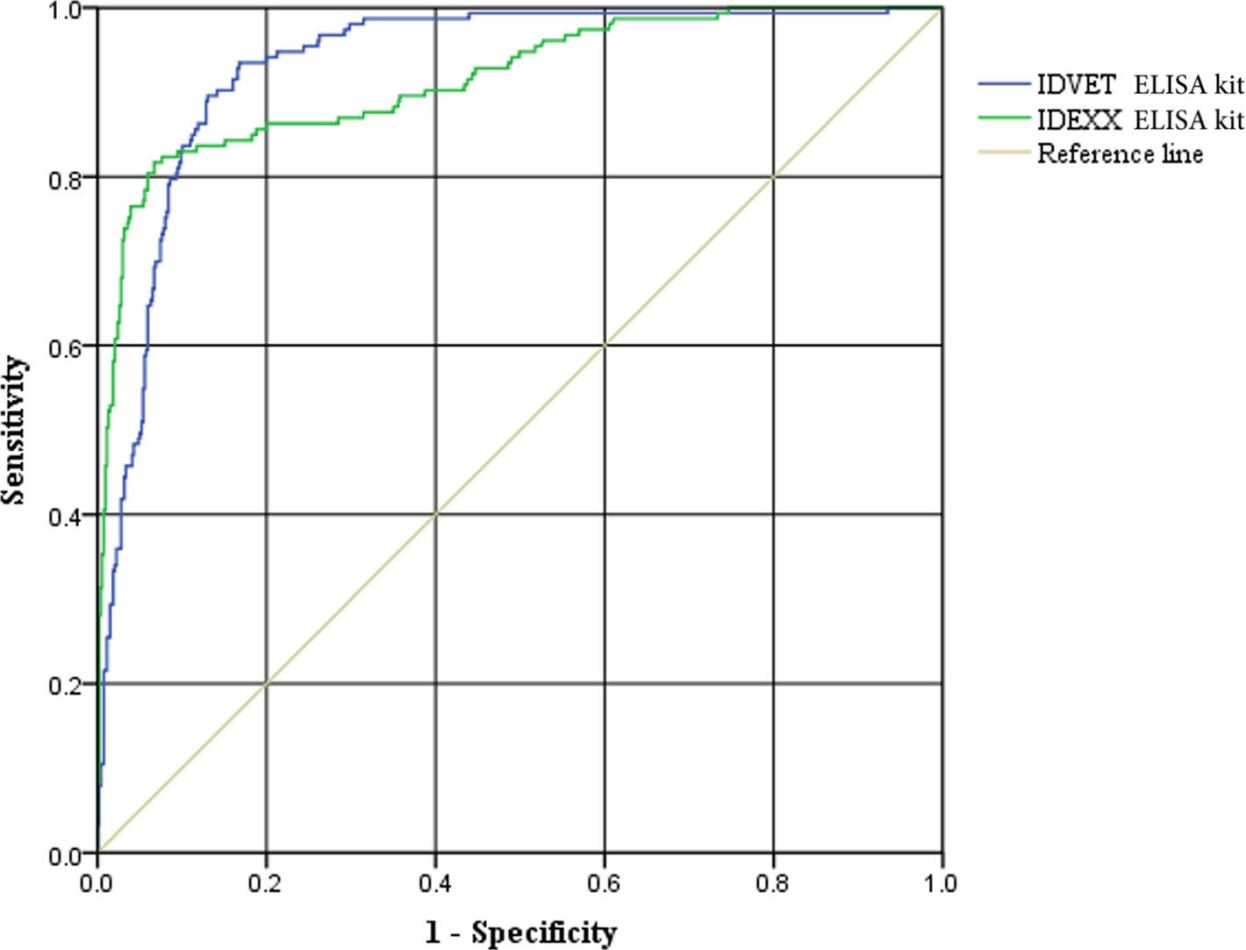
The ROC curve of the IDEXX and IDVET ELISA kit using VNT as the reference test used 690 sera

The performance of the IDEXX and IDVET ELISA kit as a diagnostic test to identify the presence of anti-AKAV antibodies was further assessed using ROC analysis (Fig. 2). The area under the curve (AUC) of the IDEXX ELISA kit was 0.915 (95% confidence interval [CI]: 0.887, 0.943), which indicated good performance for anti-AKAV antibody detection with the cut-off point of 29.29%, consistent with the S/*P* cut-off value of 30% proposed by the manufacturer. The AUC of the IDVET ELISA kit was 0.932 (95% CI: 0.912, 0.953) with the cut-off point of 38.74%, almost identical to the cut-off of 40% recommended by the manufacturer.

## Discussion

As globalization of trade continues to develop, the occurrence of animal import and export between countries is becoming more frequent. It is of extreme importance to prove to trading partners that products from the exporting country are free of virus, especial when the country of import does not have the disease(s) of concern. To elucidate the degree of AKAV infection, multiple ELISAs have been established for the detection of AKAV infection. In this study, two commonly used commercial ELISA kits were evaluated for the characteristics of detection antibodies against AKAV in cattle serum.

In our study, a total of 690 cattle serum samples were collected from Australia where AKAV infections were particularly universal in the tropical north and east coast and no SBV infection was reported [9]. The AKAV infection status of sera used in this study was determined using VNT. Diagnosis of the diseases was based on the detection of AKAV-specific antibodies by VNT or ELISA. VNT was the confirmatory test of AKAV infection and has been used in many researches [22, 23]. Although VNT was recognized as a reference assay in this study but it was not perfect, many scientists found that there could be significant cross reactivity between sera collected from animals infected with other related Orthobunyavirus genus viruses [24, 25]. VNT may also give false positive, non-specific reactivity in animals from regions that were free of these viruses. It was completely unsurprising to find that some serum samples (data not show) tested to be positive in VNT while given the negative results in both of the two ELISA tests. Given that, the assessments in our study provided measures of relative DSe and DSp compared to VNT.

The results indicated the IDVET ELISA kit showed substantially higher Dse (93.46%) and LOD in comparison with the IDEXX ELISA kit (80.39%). As the two ELISA kits were measuring antibodies to different antigens, significant variation would not be unexpected. The IDVET ELISA kit was detecting antibodies against the surface glycoprotein of AKAV, which were more likely to be involved in VNT. The IDEXX ELISA kit, coating with N protein of SBV, was detecting antibodies to a broadly reactive epitope on the nucleoprotein that was shared across many Simbu viruses [26], including AKAV. Kittelberger et al. [18] have reported that the different AKAV strains used for preparing the coating antigen of ELISAs caused difference results. It also has been found that antigenic diversity existed among AKAV isolates [27–29], and the difference between AKAV and SBV was more significant. Furthermore, differences in the characteristics of coating antigens may be responsible for difference DSe observed in the two ELISAs. Similar results have been reported by Naslund K et al [30] in SBV research, which virus was assigned to the Simbu serogroup together with AKAV. The results of those authors suggested that the ELISA kit for SBV antibodies based on whole virus antigen showed a higher sensitivity than an ELISA based on recombinant nucleoprotein. It was not completely unpredicted to find that the IDVET ELISA kit showed a slightly higher LOD in compare with the IDEXX ELISA kit. Another possible explanation for the differences in the commercial ELSA results may lie in the difference stages of viral infection in a sample. Surface glycoprotein, targeted by the IDVET ELISA kit and VNT and acted as protective antibodies by suppressing virus replication and proliferation, was generated rapidly as early as 6 days post infection [31].

Regarding to DSp, test results for the two ELISA kits were different using VNT as the reference method (*n* = 437) in our study. The DSp for the IDEXX ELISA kit was 93.48%, while the DSp was 82.31%, with 95 samples (17.69%) tested to be false positive in the IDVET ELISA kit compared with the relative gold standard. A possible reason was that the use of a purified recombinant antigen significantly decreased false-positive reactions to other proteins and increased the specificity of the assay [32–34]. Methodologically, the IDEXX ELISA kit was an indirect ELISA, while the IDVET ELISA kit was a competition ELISA. Differences in methods of the kits likely contributed to the different DSp between the two ELISA kits. In addition, different characteristics of the monoclonal antibody used in the two kits may also be responsible for this difference.

For the ROC curve, the AUC of the IDEXX and IDVET ELISA kit was 0.915 and 0.932 respectively, which displayed good performance for anti-AKAV antibody detection in the two ELISA kits [35]. The IDVET ELISA kit, which was specific for anti-AKAV antibody detection performed slightly better than the IDEXX ELISA kit, an assay able to detect antibodies against many Simbu viruses. The difference of two ELISA kits was perhaps unsurprising as the IDEXX ELISA kit was developed based on the N protein of SBV, which was expected to be good cross reactivity to other Simbu viruses, the reactivity for the detection of AKAV may not be as good as an assay using N protein based on AKAV virus.

## Conclusion

This study showed the two commonly used commercial ELISA kits tested in our study were valuable tool for AKAV diagnosis. The IDVET ELISA kit had a relative higher DSe (93.46%), but a lower DSP (82.31%). The IDEXX ELISA kit possessed a relative higher DSp (93.48%), but a lower DSe (80.39%). These results have important implications if the assays are used to screen herds or individual cattle in surveillance programs, or at border crossings for import-export inspection and quarantine.

## Methods

### Collection of serum samples

A cross-sectional study design was chosen for serum collection. Sera from a total of 690 purebred Holstein dairy cattle sourced from eight different farms in Australia were used to evaluate the performance of the ELISA kits. Seven days after imported cows entered quarantine, blood samples were collected individually. The blood was stored at 4 °C overnight to allow for serum separation and sera were pipetted into new tubes and stored at − 20 °C prior to use.

Two standard positive sera (R521and 93,124) were sourced from Elizabeth Macarthur Agricultural Institute (EMAI) and 5188 was a positive serum stored in our laboratory.

### Determination of the AKAV infection status by VNT

The infection status of all serum samples was determined using VNT, following the methods of a previously published protocol [36]. Briefly, sera were heat-inactivated at 56 °C for 30 min, then subjected to 2-fold serial dilutions to 1/128 with modified Eagle’s medium (MEM, Invitrogen, UK) supplemented with 10% fetal bovine serum (FBS, Invitrogen, UK) and mixed with an equal volume (50 μl) of MEM containing 100 tissue culture infectious dose 50 (TCID50) of AKAV (B 8935). Each dilution was plated in duplicate and incubated at 37 °C for 1 h. Next, 100 μl of 3 × 10^5^cells/mL Vero cells (Solarbio, Beijing, China) were infected with mixtures of virus–serum and incubated for 24 h at 37 °C in 5% CO_2_. After incubation, the cultures were evaluated for viral cytopathic effects (CPE) daily using an inverted microscope (LEICA 090–135.001, Solms, Germany) until day 5. Sera were considered positive if the cells were protected (no CPE observed) at a serum dilution ≥1/4 and negative at a serum dilution < 1/4.

To determine the LOD, three sera (R521, 93,124 and 5188) were tested by diluting from 1/1 to 1/256 according to the above procedure.

### Detection of anti-AKAV antibodies using the IDEXX ELISA kit

The above 690 sera and the serially diluted sera were tested for the presence of anti-AKAV antibodies using the IDEXX ELISA kit (Schmallenberg Virus Antibody Test Kit, IDEXX, Westbrook, USA). Sera dilution, incubation and washing procedures were performed strictly adhering to the manufacturer’s instructions. The optical density (OD) was measured at 450 nm using a spectrophotometer (Thermo Fisher MK3, Massachusetts, USA) within 15 min after adding stop solution. The results were interpreted using the mathematical formula provided in the manufacturer’s instructions. The kit allowed for a “grey zone” of interpretation which includes inconclusive results (S/*P* < 30%: negative; S/*P* ≥ 40%: positive and S/*P* between 30 and 40%: inconclusive). An S/*P* value of ≥30% was considered positive in our study.

### Detection of anti-AKAV antibodies using the IDVET ELISA kit

The diluted sera from 1/1 to 1/256 and all 690 sera were tested for the presence of anti-AKAV antibodies using the IDVET ELISA kit (ID Screen® Akabane Competition, ID.vet, Montpellier, France). All sera were screened for antibodies against the AKAV strictly according to the manufacturer’s instructions. The competition percentage (S/*N*) value was calculated using the sample (S) OD and dividing it by the negative control (NC) OD then multiplying by 100 ([(S) OD /(NC) OD] × 100). Similar to the IDEXX ELISA kit, a “grey zone” also existing for data interpretation (S/*N* < 30%: positive; S/*N* ≥ 40%: negative and S/*N* between 30 and 40%: inconclusive). An S/*N* value of < 40% was considered positive in our study.

### Statistical analyses

Assay performance was evaluated and the two ELISA tests were compared in terms of diagnostic sensitivity (DSe), diagnostic specificity (DSp) and kappa coefficient (κ) using VNT as the reference assay. DSe = (TP/ [TP + FN]) × 100, DSp = (TN/ [TN + FP]) × 100, κ value = (P_o_-Pe)/(1- Pe), P_o_ = (TP+ TN)/ (TP + FN + TN + FP), Pe = [TP + FN] × [TP + FP] + [TN + FP] × [TN + FN])/ (TP + FN + TN + FP)^2^. TP = number of true positives, TN = number of true negatives, FP = number of false positives and FN = number of false negatives. The κ value was calculated to test the level of agreement between the ELISA tests and VNT. The interpretation of agreement was: ≤0 = poor, 0.01–0.2 = slight, 0.21–0.4 = fair, 0.41–0.60 = moderate, 0.61–0.80 = substantial and 0.81–1 = almost perfect [37].

The test characteristics of the kit for anti-AKAV antibodies were analyzed using receiver operating characteristic curve (ROC). Statistical analyses were performed using the analytical software package SPSS Statistics 23 (IBM Corporation, Armonk, NY, USA) [36].

## Data Availability

The datasets used and/or analyzed during the current study are available from the corresponding author on reasonable request.

## Abbreviations

AKAV: Akabane virus
CPE: Cytopathic effects
DSe: Diagnostic sensitivity
DSp: Diagnostic specificity
ELISA: Enzyme-linked immunosorbent assay
FBS: Fetal bovine serum
LOD: Limit of detection
MEM: Modified Eagle’s medium
ROC: Receiver operating characteristic curve
SATV: Sathuperi virus
SBV: Schmallenberg virus
SHAV: Shamonda virus
TCID: Tissue culture infectious dose 50
VNT: Virus neutralization tests
